# Development of a new multimedia instrument to measure cancer-specific quality of life in Portuguese-speaking patients with varying literacy skills

**DOI:** 10.1186/s40064-016-2675-6

**Published:** 2016-07-04

**Authors:** Carlos Eduardo Paiva, Felipe Augusto Ferreira Siquelli, Gabriela Rossi Zaia, Diocésio Alves Pinto de Andrade, Marcos Aristoteles Borges, Alexandre A. Jácome, Gisele Augusta Sousa Nascimento Giroldo, Henrique Amorim Santos, Elizabeth A. Hahn, Gilberto Uemura, Bianca Sakamoto Ribeiro Paiva

**Affiliations:** Divisão de Mama e Ginecologia, Departamento de Oncologia Clínica, Barretos Cancer Hospital, Rua Antenor Duarte Vilella, 1331, Bairro Dr Paulo Prata, Barretos, São Paulo CEP: 14784-400 Brazil; Health-Related Quality of Life Research Group (GPQual), Barretos Cancer Hospital, Barretos, São Paulo 14784-400 Brazil; Center for Research Support - NAP, Barretos Cancer Hospital, Barretos, São Paulo 14784-400 Brazil; Barretos School of Health Sciences, Dr. Paulo Prata - FACISB, Barretos, São Paulo 14785-002 Brazil; InORP - Oncology Institute of Ribeirão Preto, Ribeirão Prêto, São Paulo 14025-270 Brazil; Hospital das Clínicas de Botucatu, Botucatu, São Paulo 18618-970 Brazil; Department of Clinical Oncology, Mater Dei Hospital, Belo Horizonte, Minas Gerais 30140-093 Brazil; Department of Medical Social Sciences and Center for Patient-Centered Outcomes, Northwestern University Feinberg School of Medicine, Chicago, IL 60611 USA; Botucatu Medical School, Universidade Estadual Paulista -UNESP, Botucatu, São Paulo 18618-970 Brazil

**Keywords:** Quality of life, Development, Validation, Multimedia, Low literacy, IqualiV-OG-21

## Abstract

**Purpose:**

To develop and validate a new multimedia instrument to measure health-related quality of life (HRQOL) in Portuguese-speaking patients with cancer.

**Methods:**

A mixed-methods study conducted in a large Brazilian Cancer Hospital. The instrument was developed along the following sequential phases: identification of HRQOL issues through qualitative content analysis of individual interviews, evaluation of the most important items according to the patients, review of the literature, evaluation by an expert committee, and pretesting. In sequence, an exploratory factor analysis was conducted (pilot testing, *n* = 149) to reduce the number of items and to define domains and scores. The psychometric properties of the IQualiV-OG-21 were measured in a large multicentre Brazilian study (*n* = 323). A software containing multimedia resources were developed to facilitate self-administration of IQualiV-OG-21; its feasibility and patients’ preferences (“paper and pencil” vs. software) were further tested (*n* = 54).

**Results:**

An exploratory factor analysis reduced the 30-item instrument to 21 items. The IQualiV-OG-21 was divided into 6 domains: emotional, physical, existential, interpersonal relationships, functional and financial. The multicentre study confirmed that it was valid and reliable. The electronic multimedia instrument was easy to complete and acceptable to patients. Regarding preferences, 61.1 % of them preferred the electronic format in comparison with the paper and pencil format.

**Conclusions:**

The IQualiV-OG-21 is a new valid and reliable multimedia HRQOL instrument that is well-understood, even by patients with low literacy skills, and can be answered quickly. It is a useful new tool that can be translated and tested in other cultures and languages.

**Electronic supplementary material:**

The online version of this article (doi:10.1186/s40064-016-2675-6) contains supplementary material, which is available to authorized users.

## Background

The main advantage of measuring health-related quality of life (HRQOL) in oncology practice is the observation of the clinical benefit from the patient’s perspective. Most available patient-reported outcome (PRO) instruments were originally developed to be self-administered by individuals from developed countries, usually characterized by having higher levels of education than Brazilians. Of note, 75 % of Brazilians have difficulty reading and interpreting written texts (Ribeiro et al. 2002) and almost 80 % of Brazilian patients preferred that interviewers administer HRQOL assessment instruments (Brabo et al. 2006). While interviewer-administration is useful for respondents with reading difficulties, disadvantages include the costs required to hire, train and supervise interviewers, and the potential for interviewer bias (Selltiz et al. 1976). Other sources of bias include social desirability (the tendency to give a favorable picture of oneself) and acquiescent response sets (the tendency to agree/disagree with statements regardless of their content) (Edwards 1957; Crowne and Marlowe 1964).

A topic of interest is the content addressed by currently available PROs in oncology. Both most widely-used cancer-specific HRQOL instruments, i.e., the European Organization for the Research and Treatment of Cancer Quality of Life Questionnaire Core 30 (EORTC QLQ-C30) and the Functional Assessment of Cancer Therapy-General (FACT-G), do not address existential issues. Specific modules for the evaluation of spirituality needs to be further supplemented, which increases the number of items and hence the time taken to complete the assessment tools. Spirituality, religiosity and personal beliefs seems to be of utmost importance for the coping strategies against cancer and its complications in Brazil, where only 8 % of individuals are not religious (IBGE 2012). Thus, we believe that a brief multidimensional cancer-specific PRO instrument that contains items addressing existential issues should be further developed.

Our hypothesis was that our PRO instrument should have been developed from the beginning focusing on the low educated patients to be used later by multimedia programs, and not a translated PRO instrument developed in other cultures and contexts. Several PROs have been developed for computer-based assessment. However, most of them were designed for use in a “paper and pencil” form and then adapted to the electronic format. The inclusion of multimedia resources (audio and/or visual aids) is a novel concept that can be especially useful for patients with low literacy skills. A new multimedia program—the “Talking Touchscreen”—was found to be acceptable, practical and user-friendly, thus providing opportunities to measure HRQOL in English- and Spanish-speaking patients with a range of literacy skills (Hahn et al. 2004; Yost et al. 2010).

The aim of this study was to develop and validate a new multimedia PRO instrument to measure HRQOL in Portuguese-speaking patients with cancer with varying literacy skills.

## Methods

### Ethics statement

The study complied with the ethical standards of the Declaration of Helsinki and Brazilian National Health Council resolution no. 466/2012. The study was approved by the Research Ethics Committee of the Barretos Cancer Hospital (Barretos-SP, Brazil, no 100.449). The Ethics Committee at the other participating centers also approved the study. Informed consent was obtained from all individual participants included in the study.

### Phase I: Qualitative study—identification of the health-related quality of life content

#### Selection of participants

The inclusion criteria were as follows: histological diagnosis of cancer (regardless of the cancer type and current treatment), age 18 years or older, have knowledge about the diagnosis of cancer, and ability to communicate in the Brazilian Portuguese language. Patients with any uncontrolled psychiatric disease or significant cognitive dysfunction were not eligible to participate. In the Phase I, at least 5 patients should be of low education status (less than 8 years of formal education); two of them should be illiterate or with less than 2 years of education. Each subgroup (cancer survivors; systemic adjuvant or neoadjuvant treatment; systemic palliative treatment; palliative care only) should be composed of similar number of participants, in order to represent all stages of the cancer care continuum.

#### Data collection

A convenience sample of patients with cancer was recruited from the Outpatient and Inpatient Units of the Clinical Oncology and Palliative Care Departments. They were interviewed individually by two researchers with previous experience in conducting qualitative research (CEP and BSRP).

Patients answered the following eight open-ended questions: (1) How does your medical condition or your treatment interfere with your physical health?; (2) How does your medical condition or your treatment interfere with your emotional health?; (3) How does your medical condition or your treatment interfere with your social activities (going out, having fun, connecting with family and/or friends)?; (4) How does your medical condition or your treatment interfere with your finances?; (5) How does your medical condition or your treatment interfere with your religious/spiritual life?; (6) If you compare yourself today with when you were not sick, what do you think has changed in your life?; (7) What did you do before getting sick that you do not do today?; and (8) What worries you the most currently?

#### Data analyses

The interviews were recorded and transcribed *verbatim* for subsequent content analysis according to Bardin’s methodology (1994). The first step was the pre-analysis, which consisted of direct and intense contact with the material and organization of the data to meet the evaluation standards, including exhaustiveness, representativeness, homogeneity and relevance. The next step was to organize the topics according to their relevance and/or repetition (codification and categorization of the data). The transcripts were independently coded into categories by two researchers; disagreements in coding were resolved during a consensus meeting. Finally, the raw data were divided into percentages and interpreted (treatment and interpretation of the obtained results). The criterion of data saturation was used to define the sample size for this phase (Miles and Huberman 1994).

### Phase II: Identification of the most relevant items

#### Instrument development strategy

Considering the content identified in the qualitative analysis, the authors created a pool of items for each category identified in the qualitative analysis. The items were grouped into a questionnaire using 5-point Likert-type responses (“not at all important”, “slightly important”, “moderately important”, “very important” and “extremely important”).

#### Selection of participants

The inclusion criteria were as follows: histological diagnosis of cancer (regardless of the cancer type and current treatment), age 18 years or older, have knowledge about the diagnosis of cancer, and ability to communicate in the Brazilian Portuguese language. Patients with any uncontrolled psychiatric disease or significant cognitive dysfunction were not eligible to participate.

#### Data collection

The instrument was administered to a sample of patients on “paper and pencil” format in private rooms at the outpatient units. Trained research coordinators from the Center for Research Support (Barretos Cancer Hospital) conducted all the interviews.

#### Data analyses

The items with the highest percentage of “extremely important” responses and those with the highest percentage of classification as among the 10 most important items were identified; these items were retained for the next study phase.

### Phase III: Development of the instrument, pretesting and pilot testing

#### Instrument development strategy

The new instrument was developed with standardized questions that always followed the same format (starting with “how often…”) and used the same response scale (“never” to “very often”). A consensus indicated that the assessment period would be the 2 weeks immediately preceding the administration of the instrument, which was considered appropriate for patients with different stages of cancer. Considering that the ultimate goal was the development of software with audiovisual resources, an answer scale with figures was developed to facilitate the responses of patients with lower educational levels. The figures were built as squares that were filled with increasing amounts of small black spots and ranged from empty (never) to almost completely filled (very often) (see Additional file 1).

The instrument was evaluated by an expert committee that consisted of 8 members (3 clinical oncologists, 1 biologist, 3 nurses and 1 surgical oncologist), 3 of whom had experience developing and validating healthcare assessment instruments. All members independently critiqued the instrument using a form designed for the study. Two authors grouped all the reviews into a single, blinded version of the critiques that was subsequently evaluated by all committee members.

#### Selection of participants

The inclusion criteria were as follows: histological diagnosis of cancer (regardless of the cancer type and current treatment), age 18 years or older, have knowledge about the diagnosis of cancer, and ability to communicate in the Brazilian Portuguese language. Patients with any uncontrolled psychiatric disease or significant cognitive dysfunction were not eligible to participate. Pretesting was performed with 15 cancer patients who had different educational levels; at least 10 with low education level (less than 8 years of education) and among them, at least 5 with less than 2 years of education.

#### Data collection

In the pretesting, cognitive debriefing interviews were conducted by experienced interviewers using a semi-structured interview guide.

A subsequent pilot testing was performed with 149 patients with cancer recruited at the Barretos Cancer Hospital. All participants responded to the 30-item instrument in “paper and pencil” format.

#### Data analysis

Pretesting—Participants were requested to explain what they understood about each item, thus creating an assessment of the “participant’s comprehension of the item”. In addition, the patients noted whether the item was confusing or embarrassing and whether they suggested any change to the instrument. A think-aloud method with cognitive debriefing was used.

Pilot testing (reduction of items)—The instrument containing 30 items was initially submitted to an exploratory factor analysis. It was performed using principal component analysis and varimax rotation. An Eigenvalue >1 was defined to extract the factors in the exploratory phase. The adequacy of the data for the factor analysis was identified using the Kaiser–Meyer–Olkin (KMO) test and Bartlett’s test of sphericity. During the initial exploratory factor analysis, the items that had low factor loadings (<0.4) in all domains were excluded. Other exclusion criteria were a percentage of unanswered items >4 % and a correlation coefficient between items of the same domain >0.8. After the initial exclusion of items, a new exploratory factor analysis was performed to define the domains. Statistical analyses were performed using SPSS software (version 20.0, Chicago, IL, USA).

### Phase IV: Evaluation of the psychometric properties of the IQualiV-OG-21—multicentre Brazilian study

#### Selection of participants

The inclusion criteria were as follows: histological diagnosis of cancer (regardless of the cancer type and current treatment), age 18 years or older, have knowledge about the diagnosis of cancer, and ability to communicate in the Brazilian Portuguese language. Patients with any uncontrolled psychiatric disease or significant cognitive dysfunction were not eligible to participate. Patients were recruited from four different Hospitals: Barretos Cancer Hospital (Barretos-SP, Brazil), Botucatu’s Medical School (Botucatu-SP Brazil), Oncology Institute of Ribeirão Preto (Ribeirão Preto-SP, Brazil) and Mater Dei Hospital (Belo Horizonte-MG, Brazil). The first two centers belong to the Brazilian public health system, and the other two serve insured or private patients.

#### Data collection

All participants responded the IQualiV-OG-21 in “paper and pencil” format and then completed both the Functional Assessment of Cancer Therapy-General (FACT-G) and the Functional Assessment of Chronic Illness Therapy-Spiritual Well-Being scale (FACIT-sp-12). To ensure high data quality, study procedures were standardized through in-person or video conference meetings.

#### Validation instruments

The Functional Assessment of Cancer Therapy-General (FACT-G) contains 27 items on a 4-point Likert-type scale assessing four domains of “well-being” (physical, social/family, emotional and functional); higher scores indicate better HRQOL (Cella et al. 1993). It has been translated to and validated for Portuguese (Ishikawa et al. 2010).

The Functional Assessment of Chronic Illness Therapy-Spiritual Well-Being scale (FACIT-sp-12) consists of 12 items and two sub-domains of spiritual well-being (peace/meaning and faith). Higher scores indicate higher levels of spiritual well-being (Peterman et al. 2002). It has been previously validated in Brazil (Lucchetti et al. 2013).

#### Data analyses

The factor structure for the IQualiV-OG-21, which was defined in Phase III, was tested in Phase IV (multicenter study); maximum likelihood estimation was used for the confirmatory factor analysis. To test goodness of fit we used the comparative fit index (CFI) and the Tucker-Lewis Index (TLI). For both, values ≥0.95 indicate a good fit, and values >0.90 an acceptable fit (Hu and Bentler 1999). Additionally, Root Mean Square Error of Approximation (RMSEA) values below 0.08 are considered to reflect acceptable fit to the model and values smaller than 0.05 as good fit (Schermelleh-Engel et al. 2003). As a requirement, all standardized factor loadings need to be greater than 0.4 and statistically significant.

The internal consistency reliability of each subscale was assessed using Cronbach’s coefficient alpha; values above 0.70 were considered adequate (Terwee et al. 2007).

Sixty four patients from the phase IV cohort were retested after 7–14 days to measure the test–retest reliability. It was assessed using the intraclass correlation coefficient (ICC); values above 0.70 were considered adequate (Terwee et al. 2007).

Known-group validity was assessed by comparing the mean IQualiV-OG-21 subscales scores between groups of patients with Eastern Cooperative Oncology Group Performance Status (ECOG-PS) values of 0–1 versus 2–4. A *t* test was used for these comparisons. Additionally, Cohen’s effect size (ES) was calculated to indicate the magnitude of the differences between groups, defined as the difference in scores between the ECOG-PS 0–1 and 2–4 groups divided by the combined standard deviation of both groups. Effect size values of <0.2, 0.2–0.49, 0.5–0.79 and ≥0.8 were classified as negligible, small, moderate and large differences, respectively (Fayers and Machin 2007). We hypothesized that the physical, functional and overall domains would be the most discriminative. For convergent validity, the IQualiV-OG-21 scores were compared with the corresponding scores on the FACT-G and FACIT-sp-12 instruments. Correlation coefficients ≥0.4 were considered adequate (Fayers and Machin 2007).

Statistical analyses were performed using SPSS software (version 20.0, Chicago, IL, USA) and confirmatory factor analysis was accomplished with SPSS Amos version. 20.0 (version 20.0, Chicago, IL, USA). A nominal significance level (*p* < 0.05) was used to interpret results.

### Phase V: Development of the software containing the IQualiV-OG-21

#### Software development

In brief, the software was developed in Java programming language and contained audio–visual resources (recorded reading of the instructions, questions and response choices, and interactive icons). The software was designed so that even illiterate respondents could answer the instrument without the need for application by an interviewer. One item at a time is presented on the computer touchscreen. Small picture icons appear below each text element allowing patients to replay the sound if necessary, to skip to the next item without answering, and allowing return to previous items to correct errors or change answers.

### Phase VI: Comparison between “paper and pencil” and software IQualiV-OG-21 formats

#### Selection of participants

The inclusion criteria were as follows: histological diagnosis of cancer (regardless of the cancer type and current treatment), age 18 years or older, have knowledge about the diagnosis of cancer, and ability to communicate in the Brazilian Portuguese language. Patients with any uncontrolled psychiatric disease or significant cognitive dysfunction were not eligible to participate.

#### Data collection

A new sample of fifty four cancer patients answered the IQualiV-OG-21 in both “paper and pencil” and software formats. The order of administration was randomized. Between the administrations of the instruments, patients were evaluated regarding their functional literacy levels using the Multidimensional Screener of Functional Health Literacy (MSFHL). The MSFHL is a simple screening tool which provides an accurate prediction of a patient’s functional literacy status. It consists of six items that provide a score ranging from 0 to 10; scores are categorized as inadequate (0–3), marginal (4–5) and adequate (6–10) functional literacy levels (Apolinario et al. 2014). In addition, patients reported their preferences about the best format (“paper and pencil” vs. indifferent vs. software). Afterwards, the patients were classified by the interviewer regarding their ability to answer the instrument in a self-administered format.

#### Data analyses

Descriptive statistics were used to describe differences between patient’s preferences and abilities to answer the instruments.

## Results

### Phase I: Qualitative study

Data saturation was identified after the initial evaluation of 20 patients. Table 1 shows the characteristics of the patients included in this phase. The interviews lasted approximately 20 min. After the transcriptions were coded, 5 categories were identified (“physical”, “emotional”, “financial”, “social” and “existential”). The category “physical” was subdivided into 7 subcategories; the category “emotional” into 4 subcategories; the category “financial” into 2 subcategories; the category “social” into 5 subcategories; and the category “existential” into 6 subcategories. Based on these results, the investigators developed a questionnaire with 43 items to use in the next study phase.

**Table 1:** Sociodemographic and clinical characteristics of the patients included in the study

Characteristic	Phase I		Phase II		Phase III		Phase IV		Phase VI	
	*(n* = 20)		(*n* = 60)		(*n* = 149)		(*n* = 323)		(*n* = 54)	
	*n*	%	*n*	%	*n*	%	*n*	%	*n*	%
Gender										
Male	8	40.0	27	45.0	49	32.9	151	46.7	36	60.0
Female	12	60.0	33	55.0	100	67.1	172	53.3	24	40.0
Age (years)										
<40	1	5.0	12	20.0	39	26.2	45	13.9	10	16.7
40–65	16	80.0	35	58.3	91	61.1	197	61.0	41	68.3
≥65	3	15.0	13	21.7	19	12.8	81	25.1	9	15.0
Educational level (years of formal schooling)										
Illiterate	0	0	0	0	4	2.7	16	5.0	4	6.7
<8	5	25.0	23	38.3	56	37.6	145	44.9	27	45.0
8–11	6	30.0	16	26.7	26	17.4	84	26.0	24	44.0
>11	9	45.0	21	35.0	63	42.3	78	24.1	5	8.3
Family income (expressed as multiples of the minimum wage)										
≤1	3	15.0	14	25.0	29	19.7	52	17.9	23	38.3
1–2	8	40.0	11	19.6	51	34.7	63	21.7	18	30.0
2.1–3	4	20.0	12	21.4	26	17.7	49	16.9	10	16.7
>3	5	25.0	19	33.9	41	27.9	126	43.4	7	12.0
Missing	0		4		2		33		2	
Brazilian region of origin										
Southeast	14	70.0	42	70.0	106	71.1	257	79.6	43	71.7
Southern	0	0	1	1.7	1	0.7	1	0.3	0	0.0
Northern	1	5.0	4	6.7	7	4.7	22	6.8	7	11.7
Midwest	5	25.0	13	21.7	34	22.8	40	12.4	8	13.3
Northeast	0	0	0	0	1	0.7	3	0.9	2	3.3
Type of primary tumor										
Breast	5	25.0	12	20.0	53	35.6	73	22.6	5	8.3
Colorectal	4	20.0	15	25.0	25	16.8	38	11.8	3	5.0
Prostate	2	10.0	7	11.7	10	6.7	33	10.2	2	3.3
Cervix	1	5.0	3	5.0	16	10.7	19	5.9	0	0
Gastric	1	5.0	1	1.7	8	5.4	9	2.8	0	0
Lung	0	0	4	6.7	3	2.0	22	6.8	0	0
Head and Neck	0	0	4	6.7	2	1.3	33	10.2	40	66.7
Esophageal	1	5.0	1	1.7	4	2.7	7	2.2	0	0
Endometrial	0	0	0	0	2	1.3	1	0.3	0	0
Others	6	30.0	13	21.7	26	17.4	88	27.2	10	16.7
Distant metastasis										
Yes	11	55.0	31	52.0	61	41	158	48.9	15	25.0
No	9	45.0	29	48.0	88	59	165	51.1	45	75.0
Type of current treatment										
Follow-up (NED)	0	0	11	18.3	16	10.7	45	13.9	3	5.0
Curative/Adj/Neoadj systemic therapy	4	20.0	18	30.0	72	48.3	120	37.2	45	75.0
Palliative systemic therapy	10	50.0	21	35.0	56	37.6	132	40.9	10	16.7
Palliative care only	6	30.0	10	16.7	5	3.5	26	8.0	2	3.3
Place of interview										
Ambulatory	9	45.0	56	93.0	117	78.5	288	89.2	59	98.3
Hospital	11	55.0	4	7.0	32	21.5	35	10.8	1	1.7
ECOG performance status										
0–1	8	40.0	NA	–	109	73.2	220	68.8	54	90.0
2–4	12	60.0	NA	–	39	26.2	100	31.3	6	10.0
Missing							3			

*NED* no evidence of disease, *Adj* Adjuvant, *Neoadj* Neoadjuvant, *ECOG* Eastern Cooperative Oncology Group

### Phase II: Identification of the most relevant items

Sixty patients answered a questionnaire containing 43 items. The items with the highest percentage of “extremely important” responses and the percentage of times they were classified among the 10 most important were identified. From this analysis, the selected items were divided into physical symptoms (8 items), emotional problems (4 items), functionality (1 item), social problems (2 items) and existential problems (2 items), for a total of 17 items. After reviewing the literature, the authors of the instrument decided to include 8 more items that aimed to assess the following: functionality (1 item), anxiety (1 item), interpersonal relationships (2 items), fun and leisure (1 item) inner peace (1 item), irritability (1 item) and financial aspects (1 item). Table 1 shows the characteristics of the patients included in this phase.

### Phase III: Construction of the initial version of the instrument, pretesting and pilot testing

The initial version of the instrument with 25 items was evaluated for content validity by an expert committee. Two authors of the study analyzed the committee members’ critiques and decided to include 5 more items to assess the following constructs: overall assessment of HRQOL (1 item), sexuality (1 item), meaning of life (1 item), prayer (1 item) and the importance of faith in fighting the disease (1 item). Additional file 2 details the construction of the initial version of IQualiV-OG with 30 items. The new version with 30 items was again evaluated by all members of the expert committee, who considered the content adequate for an instrument that aims to measure the HRQOL of patients with general cancer. Some adjustments were made to the instrument’s statements, the presentation order of a few items and the language of 3 items that were considered confusing by patients.

A total of 180 patients were invited to participate in the pilot test; of these, 152 agreed to participate. Three cases were subsequently excluded from the statistical analyses because of screening failure (there were cases of in situ carcinomas). Table 1 shows the sociodemographic and clinical characteristics of the study participants.

The KMO and Bartlett’s test values were adequate (KMO = 0.784, p < 0.001), indicating that the sample could be subjected to factor analysis. The exploratory factor analysis of the instrument with 30 items identified 9 factors that explained 68.5 % of the total variance. Nine items were excluded because they were grouped into factors other than the one they had been originally devised for. Table 2 shows the factor loadings for the 21 items that were retained. Item 1, which assesses the frequency of pain, was grouped into the emotional factor and not into the physical factor; however, considering this item’s importance to the instrument, the authors decided to keep it in the analysis of the domain for which it was devised (the physical domain). None of the items were excluded based on the percentage of missing responses or inter-item correlations within the same factor >0.8. After 9 items were excluded, the instrument was named the “*Instrumento para avaliação da Qualidade de Vida em Oncologia Geral com 21 itens*” (IQualiV-OG-21) and was composed of 21 items divided into 6 domains: emotional (6 items), physical (5 items), existential (3 items), interpersonal relationships (2 items), functional (3 items) and financial (2 items). Physical domain included items assessing physical symptoms (pain, fatigue, nausea, etc.); otherwise, functional domain included items measuring physical functioning (ability to work, take care of itself, and need to rest). The original instrument in Portuguese and a preliminary English translation are available in Additional file 1. The scores for each domain were developed using the mean of the answered items on a normalized scale ranging from 0 to 100, with higher scores representing worse HRQOL. We considered HRQOL as a multi-dimensional concept that includes different domains related each other; additionally, a composite score would represent the global HRQOL. Thus, the overall score, which is the mean of all domains, was also created.

**Table 2:** Factor analysis of the IQualiV-OG-21 (*n* = 149)

Item	Descriptors^a^	Factors					
		Emotion	Physical	Existen	Relation	Function	Financial
6	Insomnia	*0.482*	0.064	0.129	0.280	0.126	0.281
17	Depressed	*0.652*	0.133	0.247	0.252	0.069	0.239
18	Anxious	*0.736*	−0.047	0.147	0.138	−0.064	0.251
19	Easily irritated	*0.702*	0.228	0.172	0.257	−0.095	0.023
21	Fear of worsening health	*0.588*	0.012	0.335	−0.207	0.160	0.145
22	Worry about the family’s future	*0.572*	0.032	0.055	−0.059	0.191	0.460
1	Pain	0.588	*0.189*	−0.165	0.358	0.348	−0.050
2	Weak or without energy	0.399	*0.582*	−0.045	0.197	0.219	−0.204
4	Lack of appetite	0.076	*0.742*	0.084	−0.089	0.118	0.347
5	Limited ability to taste foods	−0.061	*0.755*	0.095	0.153	0.161	0.081
7	Nauseated	0.142	*0.777*	0.117	0.062	0.009	0.121
24	Hopeless	0.203	0.111	*0.784*	0.073	0.169	0.008
25	Lost faith in God	0.144	0.068	*0.786*	0.215	−0.004	0.153
29	Life did not make sense	0.109	0.088	*0.759*	0.077	0.051	−0.009
15	Relationship problems	0.225	0.112	0.132	*0.762*	0.015	0.177
16	People distanced themselves	0.151	0.059	0.205	*0.795*	0.107	0.064
9	Needing help to get dressed, shower or eat	0.067	−0.001	0.020	−0.034	*0.821*	−0.017
10	Unable to work	0.107	0.260	0.120	0.313	*0.689*	0.318
11	Needing to lie down or sit to rest	0.058	0.407	0.243	0.043	*0.648*	0.021
12	Worried about finances	0.263	0.180	0.084	0.051	0.113	*0.773*
13	Not enough money to meet needs	0.259	0.180	0.017	0.298	−0.049	*0.744*

^a^The brief item descriptors approximate the questions asked in each item

Factor loadings are italicized when grouped in a same domain

*Emotion.* emotional, *Existen.* existential, *Relation.* interpersonal relationships, *Function.* functional

### Phase IV: Evaluation of the psychometric properties (multicentre study)

From the 379 patients invited to participate in the study, 323 agreed to participate; Barretos Cancer Hospital (*n* = 181), Botucatu’s Medical School (*n* = 49), Oncology Institute of Ribeirão Preto (*n* = 50) and Mater Dei Hospital (*n* = 43). Table 1 details the patient characteristics.

The IQualiV-OG-21 was completed in a mean (SD) of 6.0 (3.3) minutes, ranging from 1.2 to 23 min. None of the items had an unanswered percentage >4 %, values ranged from 0 to 0.9 %.

Regarding the confirmatory factor analysis, fit indices were considered good (CFI = 0.950, TLI = 0.933, RMSEA = 0.051) and all factor loadings were greater than 0.40 and statistically significant.

The domains with the lowest (most favorable) mean scores were the personal relationship (mean = 13.4) and existential domains (mean = 15.2), and the domains with the highest (least favorable) scores were the emotional (mean = 41.7) and financial (mean = 39.6) domains (Table 3). Internal consistency reliability was considered adequate, with Cronbach’s alpha values ranging from 0.75 (for the personal relationship domain) to 0.90 (for the overall score; Table 3).

**Table 3:** Mean values, internal consistency and test–retest analysis of IQualiV–OG-21

IQualiV-OG-21 domains	Number of items	Mean (SD)	Cronbach’s α (95 % CI)	ICC (95 % CI)^a^
Emotional	6	41.7 (24.9)	0.85 (0.82–0.87)	0.88 (0.80–0.93)
Physical	5	33.0 (23.8)	0.79 (0.75–0.82)	0.79 (0.66–0.88)
Financial	2	39.6 (32.4)	0.81 (0.77–0.85)	0.83 (0.72–0.90)
Existential	3	15.2 (22.2)	0.81 (0.77–0.84)	0.96 (0.94–0.98)
Functional	3	28.9 (25.9)	0.77 (0.72–0.81)	0.86 (0.77–0.92)
Personal relationships	2	13.4 (20.7)	0.75 (0.69–0.80)	0.94 (0.90–0.97)
Total score	21	28.7 (17.7)	0.90 (0.89–0.92)	0.95 (0.92–0.97)

*SD* standard deviation, *ICC* intraclass correlation coefficient

^a^n = 64

Test–retest reliability was assessed in 64 participants using the ICC and was considered adequate in all domains, ranging from 0.79 (physical domain) to 0.96 (existential domain; Table 3).

Convergent validity was confirmed by the Pearson correlation between the IQualiV-OG-21 domains and the corresponding domains of the FACT-G and FACIT-sp-12 instruments. All expected correlations had coefficients greater than 0.4, with only two exceptions: personal relationship and FACT-G social (*r* = −0.242), and existential and FACIT-sp 12 faith (*r* = −0.315) (Table 4).

**Table 4:** Pearson correlations between IQualiV-OG-21 and FACT-G and FACIT-sp-12 (*n* = 323)

IQualiV-OG-21	FACT-G					FACIT-sp-12		
	Physical	Emotional	Functional	Social	Total	Meaning/peace	Faith	Spirituality total
Emotional	−0.55*	−0.63*	−0.40*	−0.07	−0.53*	−0.29*	−0.01	−0.24*
Physical	−0.72*	−0.47*	−0.47*	−0.17*	−0.61*	−0.41*	−0.25*	−0.38*
Financial	−0.33*	−0.31*	−0.27*	−0.09	−0.33*	−0.21*	−0.06	−0.17*
Existential	−0.51*	−0.60*	−0.48*	−0.18*	−0.57*	−0.48*	−0.32*	−0.46*
Functional	−0.70*	−0.45*	−0.61*	−0.21*	−0.65*	−0.44*	−0.28*	−0.41*
Personal relationships	−0.22*	−0.26*	−0.19*	−0.24*	−0.29*	−0.29*	−0.15*	−0.26*
Total score	−0.71*	−0.63*	−0.57*	−0.22*	−0.69*	−0.49*	−0.26*	−0.44*

* p < 0.01.  Underline indicates a pair of scales that should correlate theoretically

Known-groups validity was assessed by comparing the IQualiV-OG-21 scores of patients who presented better (ECOG-PS of 0-1) and worse (ECOG-PS of 2-4) functional performances. As expected, the magnitude of the difference was greatest in the following domains: physical (ES = 0.95), functional (ES = 1.16) and overall score (ES = 0.90; Table 5).

**Table 5:** Known-group validation according to the performance status

IQualiV-OG-21 domains	ECOG performance status		*P* ^a^	∆	ES
	Mean (SD)				
	0–1	2–4			
	(n = 220)	(n = 100)			
Emotional	37.6 (24.7)	50.9 (23.3)	<0.001	13.22	0.53
Physical	25.9 (22.0)	48.6 (20.2)	<0.001	22.71	0.95
Financial	33.7 (31.8)	51.6 (30.5)	<0.001	17.87	0.55
Existential	11.6 (20.6)	23.4 (23.5)	<0.001	11.87	0.53
Functional	19.6 (20.4)	49.8 (24.7)	<0.001	30.27	1.16
Personal relationships	13.5 (20.8)	13.4 (20.8)	0.952	−0.14	−0.00
Total score	23.6 (16.3)	39.6 (15.6)	<0.001	17.67	0.90

*SD* standard deviation, *∆* difference between the two groups, *ES* effect size

^a^ *T* test

### Phase VI: Comparison between “paper and pencil” and software IQualiV-OG-21 formats

Among the 54 participants in the method comparison sub-study, there were 29 (53.7 %), 9 (16.7 %) and 16 (29.6 %) patients classified as inadequate, marginal and adequate functional literacy levels, respectively, according to the Multidimensional Screener of Functional Health Literacy. Twelve (n = 12, 22.2 %; inadequate functional literacy = 10, marginal functional literacy = 2) participants required the application of the IQualiV-OG-21 in “paper and pencil” form by the interviewer. Interestingly, fifteen (n = 15, 27.8 %; inadequate functional literacy = 9, marginal functional literacy = 3, adequate functional literacy = 3) participants initially tried to self-administer the instrument in “paper and pencil” form, nevertheless they were unable to complete it; the application by the interviewer were also required. All participants were able to self-administer the electronic IQualiV-OG-21 and only 4 (7.4 %) patients required significant help by the interviewer. Regarding their preferences about the best format, 6 (11.1 %) patients preferred the “paper and pencil” format, 15 (27.8 %) reported both were equal, and 33 (61.1 %) preferred the electronic format.

## Discussion

### Principal findings

In this study, we present the first cancer-specific HRQOL instrument developed as a multimedia electronic tool for Portuguese-speaking patients. The IQualiV-OG-21 demonstrated good psychometric properties, and its electronic form was practical and well accepted by patients with cancer. By overcoming barriers to self-administration, this new instrument provides greater opportunities to measure HRQOL in patients with a range of literacy skills. Our findings are in accordance with previous studies conducted in patients with low literacy skills from Spanish- and Chinese-speaking populations (Hahn et al. 2003, 2004, 2007; Hahn and Cella 2003; Thumboo et al. 2006).

### Limitations and strengths of the study

The study has some limitations. We enrolled only ambulatory cancer patients who were well enough to participate. Results may not be generalizable to patients with greater disease severity and poorer HRQOL. The personal relationship domain of the IQualiV-OG-21 was weakly associated with the social domain of the FACT-G. This may be due to the fact that the social domain of the FACT-G measures a broader concept than our tool which is focused on relationship quality. Future studies should correlate the personal relationship domain with other instruments that more closely measure similar concepts. Similarly, the lack of a standard instrument that can be used to correlate the financial domain scores of the IQualiV-OG-21 is another limitation of that could be addressed in future studies. It might be useful to consider other psychometric approaches to scoring, such as item response theory (Van der Linden and Hambleton 1997) or the Rasch model (Bond and Fox 2007). Another limitation is that the IQualiV was not developed and tested initially as software format, but in “paper and pencil” form. The software was developed throughout the initial phases of the study, therefore, unfortunately, it was considered ready for use only in the Phase VI. Further data are needed about the software in a large cohort of patients with cancer. Moreover, the comparison between IQualiV-OG-21 software and other standard similar questionnaires is awaited.

In terms of strengths, we can point out the originality of the study, since only a handful of multimedia-containing HRQOL instruments have been developed so far. Another strength is the external validation in a multicentre cohort comprising patients from different social and educational skills. Furthermore, the IQualiV-OG-21 includes a specific existential domain, which seems to be relevant for its use when evaluating patients with cancer, since spirituality and religiosity are commonly used as coping strategies by Brazilians (Paiva et al. 2011). The widely-used PRO instruments EORTC QLQ-C30 and FACT-G do not evaluate existential aspects and thus require the administration of specific supplements for this purpose.

### Implications for clinicians and researchers

Brazil is a continental country, with about 200 million inhabitants and 576 thousand new cases of cancer each year (INCA 2014; IBGE 2015). HRQOL measurement has practical implications for use in cancer care, such as improving communication, screening symptoms and triggering protocols, monitoring responses, facilitating shared decision making, etc. (Hearn and Higginson 1997; Higginson and Carr 2001; Greenhalgh 2009). In addition, considering the large number of Brazilian patients with low literacy skills, the electronic IQualiV-OG-21 is a useful new instrument for routine practice and research. Responses are automatically recorded in spreadsheets and there will be cost saving because of the lack of need of typists and trained interviewers. Currently, we are leading the ProQualiV program (*Programa de Qualidade de Vida como um Indicador Vital*) aiming to establish the measurement of HRQOL as a routine vital indicator in a large comprehensive cancer hospital in Brazil; electronic IQualiV-OG-21 is an ideal tool to be used in this program.

### Unanswered questions and future research

An unanswered question is the lack of assessment of the IQualiV-OG-21’s responsiveness, which is considered important for its future practical use in longitudinal assessments of patients with cancer (Reeve et al. 2013). Future evaluation of minimal clinically important differences is also needed. Although the electronic IQualiV-OG-21 is user-friendly, acceptable, valid and reliable in Brazil, it should be translated and tested in other cultures and languages. To be useful in practice, an instrument needs not only to have good psychometric properties, but it also needs to have equivalent linguistic versions in several populations to be useful in international clinical trials.

## Conclusions

We present the first electronic PRO multimedia instrument for oncology developed to measure the HRQOL of Brazilian patients with cancer. The IQualiV-OG-21 can be considered well-valid, and reliable. The electronic form is acceptable and easy to complete, even by patients with low literacy skills.

## Additional files

10.1186/s40064-016-2675-6 Original instrument in Portuguese and a preliminary English translation
10.1186/s40064-016-2675-6 Detailed description of the construction of the initial version of IQualiV-OG with 30 items.
