# Supplementary material for: Development of a new multimedia instrument to measure cancer-specific quality of life in Portuguese-speaking patients with varying literacy skills
Source: Springerplus. 2016 Jul 4;5(1):972. doi: 10.1186/s40064-016-2675-6 (PMC4932016; doi:10.1186/s40064-016-2675-6)
Supplement: Supplementary file 1 — 10.1186/s40064-016-2675-6 Original instrument in Portuguese and a preliminary English translation [file 40064_2016_2675_MOESM1_ESM.pdf]

Additional file 1

IQualiV – Onco Geral-21

Data: __/__/____   Iniciais do nome: ____________   Registro hospitalar: _____________

( ) Auto aplicado   ( ) Administrado pelo entrevistador   Tempo para ser respondido:______

Este questionário tem como objetivo avaliar a sua **Qualidade de Vida**. Pense na sua vida e nas transformações nela ocorridas e responda as 21 questões abaixo. As respostas poderão variar entre 0 (nenhuma vez) a 4 (muitíssimas vezes). Será necessário que responda às questões circulando-as ou marcando um “x”.

Para responder, considere sempre as suas ÚLTIMAS DUAS SEMANAS

	Nenhuma vez	Raramente	Algumas vezes	Muitas vezes	Muitíssimas vezes
	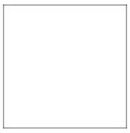	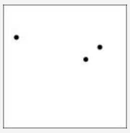	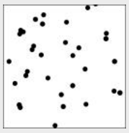	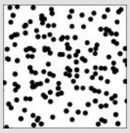	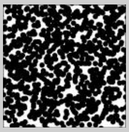
1. Com que frequência você teve dor?	**0**	**1**	**2**	**3**	**4**
2. Com que frequência sentiu-se fraco (a) ou sem energia?	**0**	**1**	**2**	**3**	**4**
3. Com que frequência você sentiu falta de apetite?	**0**	**1**	**2**	**3**	**4**
4. Com que frequência você teve dificuldades em sentir o sabor dos alimentos?	**0**	**1**	**2**	**3**	**4**
5. Com que frequência você teve enjoos (náuseas)?	**0**	**1**	**2**	**3**	**4**
6. Com que frequência precisou de ajuda para se vestir, tomar banho ou se alimentar?	**0**	**1**	**2**	**3**	**4**
7. Com que frequência sentiu-se **incapaz** de trabalhar ou de realizar o seu trabalho em casa?	**0**	**1**	**2**	**3**	**4**
8. Com que frequência precisou ficar deitado (a) ou sentado (a) para descansar?	**0**	**1**	**2**	**3**	**4**
9. Com que frequência você se sentiu preocupado (a) com a sua condição financeira?	**0**	**1**	**2**	**3**	**4**
10. Com que frequência o seu dinheiro **não foi suficiente** para satisfazer as suas necessidades?	**0**	**1**	**2**	**3**	**4**

Para responder, considere sempre as suas ÚLTIMAS DUAS SEMANAS

	Nenhuma vez	Raramente	Algumas vezes	Muitas vezes	Muitíssimas vezes
	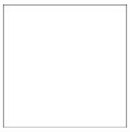	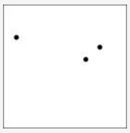	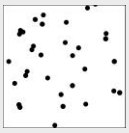	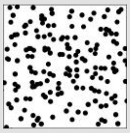	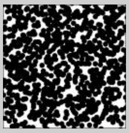
11. Com que frequência sentiu-se deprimido (a)?	**0**	**1**	**2**	**3**	**4**
12. Com que frequência sentiu-se ansioso (a)?	**0**	**1**	**2**	**3**	**4**
13. Com que frequência sentiu-se irritado (a) facilmente	**0**	**1**	**2**	**3**	**4**
14. Com que frequência você sentiu medo de que sua saúde piore?	**0**	**1**	**2**	**3**	**4**
15. Com que frequência sentiu-se preocupado (a) com o futuro da sua família?	**0**	**1**	**2**	**3**	**4**
16. Com que frequência você teve insônia?	**0**	**1**	**2**	**3**	**4**
17. Com que frequência sentiu-se **sem esperança** com a vida?	**0**	**1**	**2**	**3**	**4**
18. Com que frequência você pensou que a sua vida **não** fazia sentido?	**0**	**1**	**2**	**3**	**4**
19. Com que frequência você sentiu que **perdeu** a fé em Deus ou em algo superior?<br>□ *não se aplica, pois não acredita em Deus ou em algo superior*	**0**	**1**	**2**	**3**	**4**

Pense nas pessoas de quem gosta, aquelas que são importantes para você e responda as duas questões abaixo.

20. Com que frequência você teve problemas de relacionamento com elas?	**0**	**1**	**2**	**3**	**4**
21. Com que frequência sentiu que elas se afastaram de você?	**0**	**1**	**2**	**3**	**4**

IQualiV – Onco Geral -21

Date: __/__/____   Name initials: _____________   Hospital ID: ______________
( ) Self administered ( ) Administered by the interviewer

This questionnaire aims to assess your **Quality of Life**. Think about your life and the changes that have happened and answer the 21 questions below. The answers may vary between 0 (minimum) and 4 (maximum). Use a circle or mark an "x" to answer the questions.

To respond, consider always your LAST TWO WEEKS.

	Never	Rarely	Sometimes	Often	Very often
	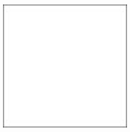	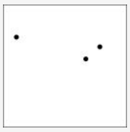	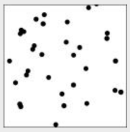	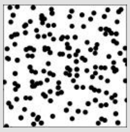	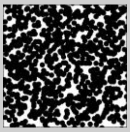
1. How often have you had pain?	0	1	2	3	4
2. How often have you felt weak and without energy?	0	1	2	3	4
3. How often have you felt a lack of appetite?	0	1	2	3	4
4. How often have you had difficulty tasting food?	0	1	2	3	4
5. How often have you had nausea?	0	1	2	3	4
6. How often have you needed help to get dressed, take a shower or eat?	0	1	2	3	4
7. How often have you felt **unable** to work or do your chores at home?	0	1	2	3	4
8. How often have you needed to lie down or sit down to rest?	0	1	2	3	4
9. How often have you worried about your finances?	0	1	2	3	4
10. How often did you have **not enough** money to meet your needs?	0	1	2	3	4

To respond, consider always your LAST TWO WEEKS.

	Never	Rarely	Sometimes	Often	Very often
11. How often have you felt depressed?	**0**	**1**	**2**	**3**	**4**
12. How often have you felt anxious?	**0**	**1**	**2**	**3**	**4**
13. How often have you felt easily irritated?	**0**	**1**	**2**	**3**	**4**
14. How often have you worried that your health will get worse?	**0**	**1**	**2**	**3**	**4**
15. How often have you worried about the future of your family?	**0**	**1**	**2**	**3**	**4**
16. How often have you had insomnia?	**0**	**1**	**2**	**3**	**4**
17. How often have you felt **hopeless** about life?	**0**	**1**	**2**	**3**	**4**
18. How often have you thought that your life did not make sense?	**0**	**1**	**2**	**3**	**4**
19. How often has your faith in God or a higher power helped you face your illness? □ *not applicable, (no belief in God or a higher power)*	**0**	**1**	**2**	**3**	**4**

Think about the people you care about, those who are important to you, and answer the two questions below.

20. How often have you had relationship problems with them?	**0**	**1**	**2**	**3**	**4**
21. How often have you felt that they distanced themselves from you?	**0**	**1**	**2**	**3**	**4**

IQualiV – Onco Geral-21, version 1.2 – for use, it is necessary to obtain permission from the author (Carlos E. Paiva; E-mail: caredupai@gmail.com)
