# Supplementary material for: Development of a new multimedia instrument to measure cancer-specific quality of life in Portuguese-speaking patients with varying literacy skills
Source: Springerplus. 2016 Jul 4;5(1):972. doi: 10.1186/s40064-016-2675-6 (PMC4932016; doi:10.1186/s40064-016-2675-6)
Supplement: Supplementary file 2 — 10.1186/s40064-016-2675-6 Detailed description of the construction of the initial version of IQualiV-OG with 30 items. [file 40064_2016_2675_MOESM2_ESM.pdf]

Additional File 2. Detailed description of the construction of the initial version of IQualiV-OG with 30 items.

Item no	Item	Phase were the item was added		
		QR	LR	EC
**1**	How often have you had pain?	X[a]		
**2**	How often have you felt weak and without energy?	X[a]		
**3**	How often have you had breathlessness?	X[a]		
**4**	How often have you felt a lack of appetite?	X[a]		
**5**	How often have you had difficulty tasting food?	X[a]		
**6**	How often have you had insomnia?	X[a]		
**7**	How often have you had nausea?	X[a]		
**8**	How often have you had constipation?	X[a]		
**9**	How often have you needed help to get dressed, take a shower or eat?	X[c]		
**10**	How often have you felt unable to work or do your chores at home?	X[d]		
**11**	How often have you needed to lie down or sit down to rest?		X[c]	
**12**	How often have you worried about your finances?	X[d]		
**13**	How often did you have not enough money to meet your needs?		X[d]	
**14**	How often have you had fun or leisure activities?		X[d]	
**15**	Think about the people you care about, those who are important to you. How often have you had relationship problems with them?		X[d]	
**16**	Think about the people you care about, those who are important to you. How often have you felt that they distanced themselves from you?		X[d]	
**17**	How often have you felt depressed?	X[b]		
**18**	How often have you felt anxious?		X[b]	
**19**	How often have you felt easily irritated?		X[b]	
**20**	How often have you missed your home or your family's relationship?	X[b]		
**21**	How often have you worried that your health will get worse?	X[b]		
**22**	How often have you worried about the future of your family?	X[b]		
**23**	How often have you felt satisfied with your sex life?			X[d]
**24**	How often have you felt hopeless about life?	X[e]		
**25**	How often have you felt that you lost your faith in God or in a higher power?	X[e]		
**26**	How often has your faith in God or a higher power helped you face your illness?			X[e]

**27**	How often did you felt inner peace?		X[e]	
**28**	How often have you prayed for the improvement of your health?			X[e]
**29**	How often have you thought that your life did not make sense?			X[e]
**30**	In general, how would you rate your quality of life?			X

Legend: QR=Qualitative research; LR=literature review; EC=expert committee.

[a]Physical domain; [b]emotional domain; [c]functional domain; [d]social domain; and [e]existential domain.
